# The time between symptom onset and various clinical outcomes: a statistical analysis of MERS-CoV patients in Saudi Arabia

**DOI:** 10.1098/rsos.240094

**Published:** 2024-11-20

**Authors:** Yehya M. Althobaity, Muhammad H. Alkhudaydi, Edward M. Hill, Robin N. Thompson, Michael J. Tildesley

**Affiliations:** ^1^ Department of Mathematics, Taif University, Taif 11099, Saudi Arabia; ^2^ The Zeeman Institute for Systems Biology and Infectious Disease Epidemiology Research, School of Life Sciences and Mathematics Institute, University of Warwick, Coventry CV4 7AL, UK; ^3^ Mathematical Institute, University of Oxford, Oxford OX2 6GG, UK

**Keywords:** delay distribution, MERS-CoV, statistical analysis, mathematical modelling

## Abstract

In this study, we investigate the impact of demographic characteristics on Middle East respiratory syndrome coronavirus (MERS-CoV) cases in Saudi Arabia, specifically focusing on the time intervals between symptom onset and key events such as hospitalization, case confirmation, reporting and death. We estimate these intervals using data from 2196 cases occurring between June 2012 and January 2020, partitioning the data into four age groups (0–24 years, 25–49 years, 50–74 years and 75–100 years). The duration from symptom onset to hospitalization varies between age cohorts, ranging from 4.03 to 4.75 days, with the 75–100 age group experiencing the longest delay. The interval from symptom onset to case confirmation spans 5.83–8.24 days, and again, the 75–100 age group faces the lengthiest delay. The interval from symptom onset and case reporting ranges from 7.0 to 9.8 days, with the 75–100 age group experiencing the longest delay. The period from symptom onset to death varies across age groups (12.3–16.1 days), with elevated mortality rates during outbreaks. Importantly, we observe age-based differences in the risk of hospitalization and other measures of infection severity, including the probability of death conditional on hospitalization. Careful quantification of epidemiological characteristics, including inference of key epidemiological periods and assessments of differences between cases of different ages, plays a crucial role in understanding the progression of MERS-CoV outbreaks and formulating effective public health strategies to mitigate their impact.

## 1. Introduction

Middle East respiratory syndrome coronavirus (MERS-CoV) is a viral pathogen that was first identified in 2012. The virus belongs to the family of coronaviruses [1], and the first MERS-CoV cases were reported in Saudi Arabia. As of January 2020, the cumulative global incidence of MERS-CoV comprised 2519 laboratory-confirmed cases, including 866 deaths, equating to a case-fatality rate of 34.3%. The majority of these cases have occurred in Saudi Arabia, totalling 2196, accompanied by 788 associated deaths, representing a case-fatality rate of 38.1% [2]. The virus is primarily transmitted to humans through close contact with dromedary camels, which serve as the reservoir host [3]. However, human-to-human transmission also occurs [4], with a particularly high level of transmission observed in healthcare settings. Among the 1379 MERS-CoV cases documented during the study period of the article by Adegboye *et al*. [5], 321 cases (23.3%) were linked to hospital infection, with 203 cases (14.7%) specifically affecting healthcare workers.

The time elapsed between the onset of symptoms and admission to the hospital stands as a significant factor influencing the prognosis of MERS-CoV. This delay can arise from various factors, including individual characteristics, healthcare system infrastructure and the severity of the illness. Age emerges as a particularly influential determinant, impacting both the promptness of seeking medical attention and the overall course of the disease. Referred to as prehospital delay, the period preceding hospitalization is commonly evaluated in individuals exhibiting MERS-CoV symptoms to assess treatment trajectories [6]. Younger individuals without underlying health conditions may overlook initial signs of MERS-CoV and undervalue the importance of seeking medical assistance. This phenomenon could be because initial symptoms of MERS-CoV infection are non-specific or could be owing to the widespread dissemination of information, particularly through social media, regarding the higher morbidity and mortality rates of MERS-CoV in older populations. Consequently, adolescents and young adults may perceive a lower personal risk of MERS-CoV compared with older individuals, leading to delayed healthcare-seeking behaviour [7].

Timely diagnosis of MERS-CoV poses a significant obstacle for global healthcare systems, leading to transmission clusters within both communities and healthcare settings [8,9]. Despite a high level of clinical suspicion surrounding MERS-CoV cases [1,10], many patients experience delays in obtaining a diagnosis and seeking prompt medical attention [11]. In addition, a considerable gap between the onset of suspected clinical symptoms and laboratory confirmation of MERS-CoV highlights the persistent challenge in achieving timely detection and intervention, necessitating improved diagnostic strategies and an enhanced healthcare response.

The time from symptom onset to confirmation affects the interval from symptom onset to discharge substantially, with age playing a crucial role in this relationship. Specifically, a shorter duration from symptom onset to confirmation correlates with a reduced time from symptom onset to discharge. Early diagnosis of MERS-CoV could potentially improve the management of symptoms and disease progression, leading to fewer severe cases and improving the availability of hospital beds for the most urgent cases. Effective resource management is particularly important in locations with limited medical facilities. In such circumstances, expediting patient treatment and discharge is paramount for effective disease control. From a clinical perspective, reducing the treatment duration stands as a key strategy for enhancing patient safety, improving quality of life, ensuring healthcare staff safety and alleviating staff workload [12].

Efficient case isolation has the potential to limit the impact of outbreaks. The efficacy of case isolation is notably influenced by the interval between symptom onset and a confirmed diagnosis or report. A shorter reporting delay substantially diminishes the transmission risk, whereas a prolonged delay hampers outbreak containment efforts and elevates the effective reproduction number.

The time delay from the onset of illness to death can be important for estimating the case fatality ratio [13]. Factors unique to each individual, such as age and existing health conditions, may potentially account for variations in the length of hospital stays [2]. The high mortality rate associated with MERS-CoV compared with the number of confirmed cases remains a concern, underscoring the importance of continued surveillance and research efforts to better understand and control this emerging infectious disease [14].

Currently, there is a limited understanding regarding the time between the onset of symptoms and various stages of MERS-CoV cases, including hospital admission, confirmation, reporting and death, specifically categorized by age groups in Saudi Arabia. Nonetheless, having information regarding the duration of hospital stays is crucial for predicting the required number of hospital beds, including both general beds and those in the intensive care unit, as well as monitoring the strain on healthcare facilities [7].

Gaining a comprehensive understanding of the different sources of delays in diagnosis and seeking medical care for MERS-CoV infection is crucial for improving the diagnostic process. By doing so, we can reduce transmission and optimize medical care, highlighting the significance of addressing these issues as essential efforts in combating the disease [2].

In this article, we estimate the time periods between symptom onset in MERS-CoV cases and key clinical outcomes, such as hospitalization, confirmation, reporting and death. The period from symptom onset to reporting reflects the delay in registering cases in Ministry of Health (MoH) records, which in turn affects the time period before cases are reported in the public domain after confirmation. Through a comprehensive analysis using various parametric distributions, we scrutinize and compare time intervals characterizing these events. Our approach provides a detailed analysis of epidemiological data, and we explain the statistical methods used to estimate the parameters of delay distributions. We also investigate the probability of death given hospitalization. Following this methodical groundwork, we present specific findings, providing a nuanced understanding of the characteristics of MERS-CoV cases and identifying promising avenues for future research.

## 2. Methods

### 2.1. Analysis of the time delay from symptom onset to clinical outcomes in MERS-CoV patients

**Figure 1.**
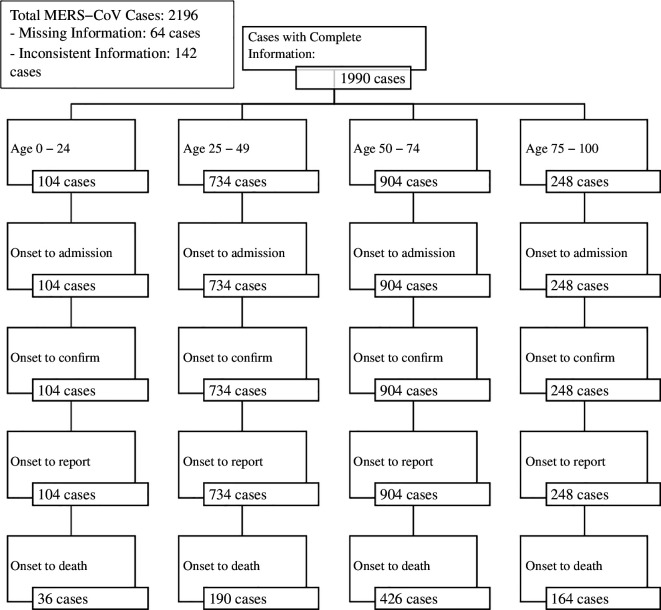
The distribution of MERS-CoV cases across different age groups from symptom onset to death, showcasing the number of cases analysed for each age category.

Comprehensive data on MERS-CoV infections are available from hospitalized patients reported in the Saudi Arabia clinical database, with reporting mandated by the MoH. This registry includes detailed information on the dates of patient admission, infection confirmation and reporting to health authorities, as well as outcomes (recovery or death). The data cover clinical symptoms and laboratory results, providing valuable insights into disease presentation. Among 2196 hospitalizations in the database, 64 cases had missing information and 142 had inconsistencies in time intervals between symptom onset and clinical outcomes. Some cases had over 30 days between admission and confirmation, highlighting the need for stringent exclusion criteria. The age distribution of MERS-CoV cases included in the study is shown in figure 1.

**Figure 2.**
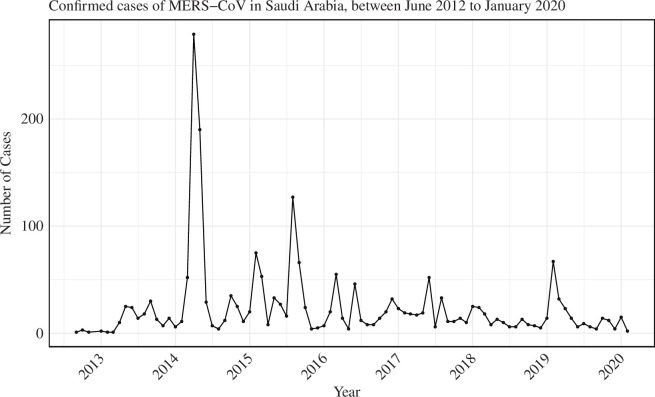
Monthly number of confirmed MERS-CoV cases in Saudi Arabia from June 2012 to January 2020.

Patients were classified into four age groups: children and young adults (0–24 years), working-age (25–49 years), seniors (50–74 years) and elderly (over 75 years). These groupings were derived from individual ages in the dataset. For reliability, we identified cases with comprehensive clinical outcomes, including hospitalization, confirmation, reporting and recovery or death, and excluded cases with missing information or inconsistent timelines. In our analysis, the dataset covered a long time period so that the effect of right truncation would be expected to be negligible. Figure 2 illustrates the distribution of cases in Saudi Arabia from June 2012 to January 2020.

We used three distinct parametric models to assess the time delay distributions from symptom onset to various clinical outcomes. These models were gamma, Weibull and lognormal. These models are commonly used for variables with non-negative values, offering a comprehensive framework for describing delay distributions for events such as admission, confirmation, reporting and either recovery or death [15]. Considering the recorded event times are specified in days, we account for the discrete nature of the data and employ the exact method to estimate the time from onset to other clinical outcomes, as in [16]. In our analysis, event i represents the date of symptom onset for a MERS-CoV patient, with progression through various clinical stages such as hospitalization, confirmation, reporting and ultimately either recovery or death, denoted as event j. This progression, illustrated as i→j, depicts the transition from symptom onset (i) to the final clinical outcome (j). As noted above, we model the time differences between symptom onset and subsequent clinical milestones using gamma, lognormal and Weibull distributions, characterized by their respective probability density functions as follows:

— Gamma distribution:
$$f(t;\alpha ,\beta )=\frac{\beta^{\alpha }}{\Gamma (\alpha )}t^{\alpha -1}e^{-\beta t},$$

where t>0,α>0 and β>0;

— Lognormal distribution:

$$f(t;\mu ,\sigma )=\frac{1}{t\sigma \sqrt{2\pi }}e^{-\frac{(ln(t)-\mu {)}^{2}}{2\sigma^{2}}},$$

where t>0, and μ and σ are the mean and standard deviation (s.d.) of the distribution, respectively.

— Weibull distribution:

$$f(t;\lambda ,k)=\frac{k}{\lambda }{\left(\frac{t}{\lambda }\right)}^{k-1}e^{-{\left(\frac{t}{\lambda }\right)}^{k}},$$

where t>0,λ>0 and k>0.

To determine the optimal parametric distribution and regression model from the set of candidate models, we employed the Akaike information criterion (AIC) [17]. The primary objective of the AIC is to balance model simplicity and fit [18]. It addresses the trade-off between underfitting and overfitting by estimating information loss and prioritizing models with minimal loss, ranking candidate models based on their AIC values, with the lowest AIC indicating the ‘best’ model out of the candidate set of models.

Additionally, we varied the date of symptom onset to compare our results with those obtained using a parametric survival analysis method that accounts for interval-censored data, as detailed in the electronic supplementary material, S2. Interval censoring describes a type of data where the exact timing of events is unknown but falls within a certain interval [4]. By using this approach, we aim to better estimate the distribution of time-to-event data and reduce potential biases associated with uncertain symptom onset dates. This comparison helps to validate the robustness of our primary analysis method.

Varying the date of symptom onset is crucial for several reasons. First, it allows us to assess the sensitivity of our results to different assumptions about symptom onset timing. This is important because the exact date of symptom onset can significantly influence the estimated intervals to subsequent events such as hospitalization, confirmation of infection and death. Second, it helps us understand the potential impact of delays in reporting and testing, which can distort the observed data. By varying the onset dates, we can account for uncertainty in delays and explore the effect of this uncertainty on our quantitative results.

Regarding the presentation of both methods, we acknowledge the importance of clarity in our approach. We included these analyses to provide a comprehensive comparison and to highlight the robustness of our primary method. Our justification for their inclusion is that they demonstrate the sensitivity of our results to different methodologies, which helps validate the reliability of our primary findings. We believe that both analyses—those that account for direct analysis and those that address interval censoring—are important for a thorough evaluation. Therefore, we will retain both approaches to ensure the robustness and validity of our conclusions.

### 2.2. Probability of death given hospitalization

To estimate the probability of death given hospitalization for each age group, we employed Bayesian inference. Bayesian inference allows for the updating of the probability distribution of a parameter based on prior beliefs and observed data. In this study, we used a Beta distribution with parameters α=1 and β=1 as the prior distribution. This choice represents a flat (non-informative) prior, meaning we started with no strong prior beliefs about the probability of death, thereby allowing the observed data to predominantly inform the posterior distribution.

The likelihood of observing k deaths out of n hospitalizations follows a binomial distribution. When combined with the flat prior, the posterior distribution for the probability of death given hospitalization is a Beta distribution with updated parameters. Specifically, the posterior parameters are:


$$\alpha_{\text{post}}=\alpha_{\text{prior}}+k,$$

$$\beta_{\text{post}}=\beta_{\text{prior}}+n-k.$$

The posterior mean, which serves as the estimated probability of death, is calculated as:


$$\text{posterior mean}=\frac{\alpha_{\text{post}}}{\alpha_{\text{post}}+\beta_{\text{post}}}.$$

To quantify the uncertainty in our estimates, we calculated 95% credible intervals (CI) from the posterior beta distribution. These intervals, derived from the 2.5th and 97.5th percentiles, provide a range within which the true probability of death is likely to fall, given the observed data and prior distribution.

### 2.3. Software and analysis tools

We conducted our data analysis and model fitting using R v. 4.1.0. The results presented in this study were obtained through the use of several R packages. Specifically, we used the ‘fitdistrplus’ package, v. 4.1.2, for model fitting and calculation of the AIC. We used the ‘tidyverse’ package (v. 2.0.0), along with the ‘dplyr’ package (v. 1.1.4), for data manipulation and visualization tasks. We accounted for interval censoring using the ‘icenReg’ package (v. 2.0.1) to generate parametric estimates of the time delay between symptom onset and various clinical outcomes.

The MERS-CoV dataset is accessible upon direct request from the MoH of Saudi Arabia (https://od.data.gov.sa/en/request-dataset). For transparency and reproducibility, the code used in this analysis is available on GitHub at https://github.com/Yehyaalthobaity/MERS-COV_daley.

## 3. Results

### 3.1. Time from symptom onset to hospitalization

**Figure 3.**
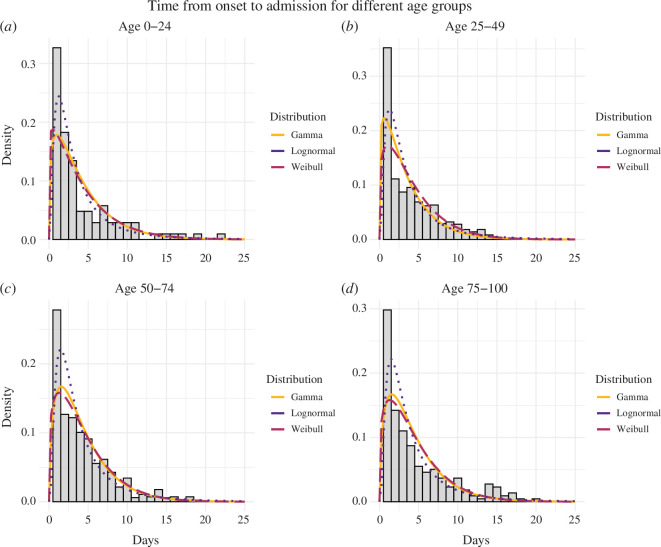
Probability distributions characterizing the observed times between symptom onset and hospitalization for four age groups ((*a*) 0–24 years, (*b*) 25–49 years, (*c*) 50–74 years and (*d*) 75–100 years, encompassing 104, 734, 904 and 248 cases, respectively). These figures represent the proportion of the population captured in the survey. A comparative analysis of the delay distribution is then conducted across these distinct age groups. The probability distributions of the gamma, Weibull and lognormal distributions illustrate the distribution of times from symptom onset to hospitalization for each specific age group.

Age significantly impacts the time from symptom onset to hospitalization. Among the youngest age group (0-24 years), the delay is notably short, with a mean of 4.03 (95% CI: 3.08–5.36) days, with patients experiencing only a minor delay. By contrast, both the 25–49 years and the 50–74 years age groups show increased delays, with mean values of approximately 4.12 (95% CI: 3.67–4.53) days and 4.35 (95% CI: 3.96–4.78) days, respectively. Elderly individuals (over 75 years) face a further increase in delay, with a mean of 4.67 (95% CI: 3.83–5.75) days. Notably, substantial variations exist in delay durations within different age groups. The lognormal distribution consistently outperforms the gamma and Weibull distributions (as shown in table 1), displaying lower AIC values across all age groups. This suggests that the lognormal distribution provides the best fit for the observed data. The fitted distributions are shown alongside the data in figure 3. Additionally, we varied the date of symptom onset to compare our main results with analogous results accounting for interval censoring, showing only a small variation as detailed in the electronic supplementary material, S2.

**Table 1.** Estimated distributions characterizing the time from symptom onset to hospitalization, for individuals of different ages. (The age groups are 0–24 years, 25–49 years, 50–74 years and 75–100 years, encompassing 104, 734, 904 and 248 cases, respectively; 95% credible intervals are shown in parentheses.)

gamma/age group	mean	shape	rate	AIC
0–24	4.25 (4.00–4.52)	1.32 (1.04–1.72)	0.31 (0.23–0.43)	505.8050
25–49	4.17 (4.00–4.21)	1.46 (1.33–1.60)	0.35 (0.32–0.40)	3362.776
50–74	4.36 (4.27–4.50)	1.57 (1.44–1.71)	0.36 (0.32–0.40)	4085.591
75–100	4.75 (4.54–4.86)	1.33 (1.12–1.59)	0.28 (0.23–0.35)	1103.379
Weibull/age group	mean	shape	scale	AIC
0–24	4.20 (3.65–4.81)	1.09 (0.94–1.29)	4.34 (3.55–5.20)	508.5948
25–49	4.08 (3.91–4.29)	1.21 (1.14–1.28)	4.35 (4.10–4.64)	3378.399
50–74	4.34 (4.16–4.54)	1.25 (1.18–1.32)	4.67 (4.41–4.94)	4109.753
75–100	4.69 (4.33–5.13)	1.14 (1.02–1.27)	4.92 (4.37–5.53)	1107.842
lognormal/age group	mean	mean-log	s.d.-log	AIC
0–24	4.03 (3.08–5.36)	1.00 (0.83–1.16)	0.89 (0.77–1.02)	486.8919
25–49	4.12 (3.67–4.53)	1.02 (0.95–1.08)	0.89 (0.84–0.93)	3307.768
50–74	4.35 (3.96–4.78)	1.11 (1.05–1.17)	0.85 (0.81–0.89)	4031.868
75–100	4.67 (3.83–5.75)	1.12 (1.00–1.24)	0.92 (0.83–1.01)	1080.411

### 3.2. Time from symptom onset to confirmation

**Figure 4.**
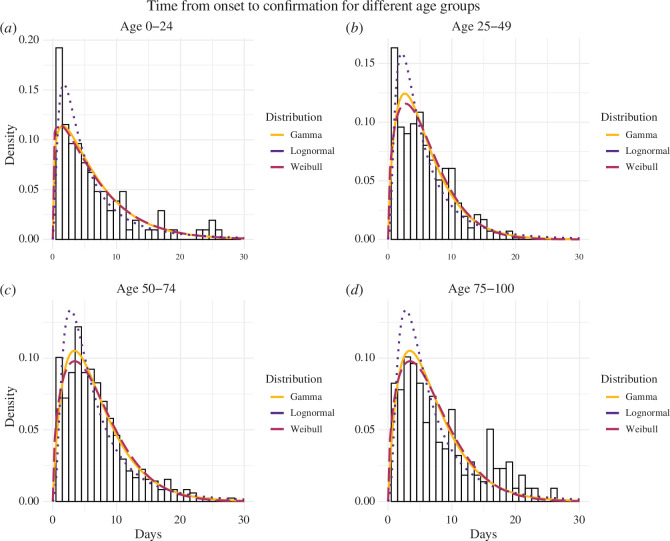
The probability density of the observed time between symptom onset and confirmation is portrayed for four age groups: (*a*) 0–24 years, (*b*) 25–49 years, (*c*) 50–74 years and (*d*) 75–100 years, encompassing 104, 734, 904 and 248 cases, respectively. These figures represent the proportion of the population captured in the survey. A comparative analysis of the delay distribution is then conducted across these distinct age groups. The probability density distributions of the gamma, Weibull and lognormal distributions illustrate the distribution of times from symptom onset to confirmation within each specific age group.

In our analysis, we observed distinct patterns in the duration between symptom onset and confirmation between age groups. For individuals aged 0−24 years, the lognormal distribution was identified as the best-fitting model based on the AIC value, with an average duration of 6.62 days (95% CI: 4.88−9.15). For individuals aged 25−49 years, the Weibull distribution proved to be the best-fitting model according to the AIC value, with an average of 5.77 days (95% CI: 5.53−6.03). Conversely, for those aged 50–74 years and 75–100 years, the gamma distribution was a better fit, with means of 7.00 (95% CI: 6.88−7.71) and 8.19 (95% CI: 8.16−8.64) days, respectively. Detailed estimates of the parameters for each distribution are presented in table 2, and the fitted probability distributions are depicted in figure 4. Furthermore, we varied the date of symptom onset to compare our main results with analogous results accounting for interval censoring, finding only a small variation in our results as detailed in the electronic supplementary material, S2.2.

**Table 2.** Estimated distributions characterizing the time from symptom onset and case confirmation, for individuals of different ages. (The age groups are 0–24 years, 25–49 years, 50–74 years and 75–100 years, encompassing 104, 734, 904 and 248 cases, respectively; 95% credible intervals are shown in parentheses.)

gamma/age group	mean	shape	rate	AIC
0–24	6.60 (6.10–7.00)	1.32 (1.05–1.71)	0.20 (0.15–0.28)	595.9135
25–49	5.83 (5.68–6.11)	1.81 (1.65–1.99)	0.31 (0.27–0.35)	3788.209
50–74	7.00 (6.88–7.71)	1.96 (1.79–2.15)	0.28 (0.26–0.32)	4753.468
75–100	8.19 (8.16–8.64)	1.72 (1.47–2.04)	0.21 (0.17–0.25)	1326.883
Weibull/age group	mean	shape	scale	AIC
0–24	6.49 (5.69–7.39)	1.14 (0.99–1.34)	6.81 (5.67–8.05)	597.6149
25–49	5.77 (5.53–6.03)	1.43 (1.36–1.52)	6.36 (6.04–6.70)	3787.632
50–74	6.84 (6.56–7.13)	1.47 (1.39–1.54)	7.56 (7.20–7.93)	4765.670
75–100	8.24 (7.62–8.98)	1.38 (1.24–1.54)	9.03 (8.17–9.98)	1327.634
lognormal/age group	mean	mean-log	s.d.-log	AIC
0–24	6.62 (4.88–9.15)	1.44 (1.25–1.62)	0.95 (0.82–1.09)	590.4715
25–49	6.01 (5.48–6.66)	1.45 (1.39–1.51)	0.83 (0.79–0.88)	3834.006
50–74	7.04 (6.48–7.72)	1.64 (1.58–1.70)	0.79 (0.76–0.83)	4801.383
75–100	8.66 (7.20–10.3)	1.79 (1.67–1.90)	0.86 (0.78–0.94)	1339.061

### 3.3. Time from symptom onset to reporting

**Figure 5.**
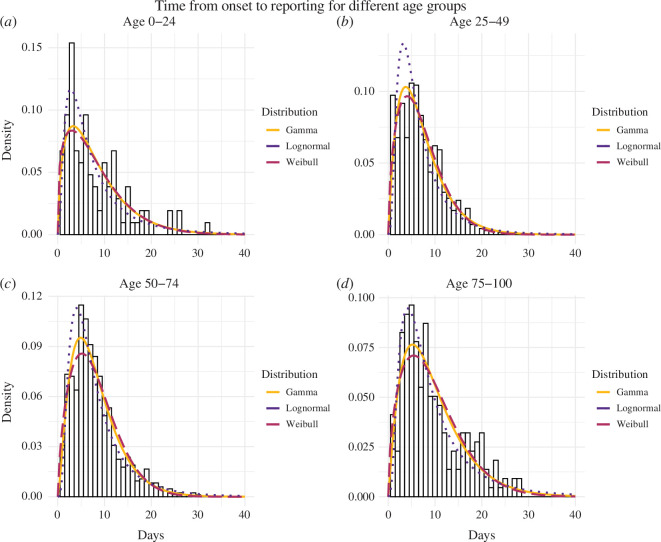
The probability density of the observed time between symptom onset and reporting is portrayed for four age groups: (*a*) 0–24 years, (*b*) 25–49 years, (*c*) 50–74 years and (*d*) 75–100 years, encompassing 104, 734, 904 and 248 cases, respectively. These figures represent the proportion of the population captured in the survey. A comparative analysis of the delay distribution is then conducted across these distinct age groups. The probability density distributions of the gamma, Weibull and lognormal distributions illustrate the distribution of times from symptom onset to confirmation within each specific age group.

We again compared fitted gamma, Weibull and lognormal distributions. For individuals aged 0–24 years, 50–74 years and 75–100 years, the gamma distribution provided the best fit, characterized by mean values of 8.04 (95% CI: 7.11−8.86) days, 8.09 (95% CI: 8.01−8.17) days and 9.81 (95% CI: 8.55−9.73), respectively. Conversely, individuals aged 25–49 years were best represented by the Weibull distribution, with means of 7.00 (95% CI: 6.70−7.32) days. These choices were substantiated by the lower AIC values compared with the lognormal distribution, confirming the appropriateness of our selections as detailed in table 3. These findings highlight the importance of understanding age-related differences in reporting timelines. The fitted distributions are shown in figure 5, and we again compared our results to analogous results using the interval censoring method, revealing small differences as detailed in the electronic supplementary material, S2.3.

**Table 3.** Estimated distributions characterizing the time from symptom onset to reporting, for individuals of different ages. (The age groups are 0–24 years, 25–49 years, 50–74 years and 75–100 years, encompassing 104, 734, 904 and 248 cases, respectively; 95% credible intervals are shown in parentheses.)

gamma/age group	mean	shape	rate	AIC
0–24	8.04 (7.11–8.86)	1.69 (1.33–2.19)	0.21 (0.15–0.27)	631.9216
25–49	7.17 (7.00–7.26)	2.08 (1.89–2.31)	0.29 (0.26–0.33)	4000.193
50–74	8.09 (8.01–8.17)	2.51 (2.29–2.75)	0.31 (0.28–0.34)	4894.916
75–100	9.81 (8.55–9.73)	2.16 (1.81–2.58)	0.22 (0.18–0.27)	1365.783
Weibull/age group	mean	shape	scale	AIC
0–24	8.06 (7.11–9.08)	1.32 (1.15–1.55)	8.76 (7.48–10.1)	634.0786
25–49	7.00 (6.70–7.32)	1.57 (1.48–1.66)	7.80 (7.41–8.20)	3993.266
50–74	8.06 (7.74–8.38)	1.66 (1.58–1.75)	9.02 (8.63–9.42)	4918.846
75–100	9.53 (8.88–10.34)	1.55 (1.42–1.72)	10.6 (9.77–11.6)	1368.161
lognormal/age group	mean	mean-log	s.d.-log	AIC
0–24	8.18 (6.41–10.81)	1.75 (1.60–1.92)	0.84 (0.72–0.96)	632.1582
25–49	7.33 (6.69–8.04)	1.68 (1.62–1.74)	0.79 (0.75–0.83)	4074.812
50–74	8.17 (7.62–8.75)	1.87 (1.82–1.91)	0.68 (0.65–0.72)	4937.991
75–100	9.78 (8.42–11.5)	2.00 (1.90–2.10)	0.75 (0.68–0.83)	1379.239

### 3.4. Time from symptom onset to death

**Figure 6.**
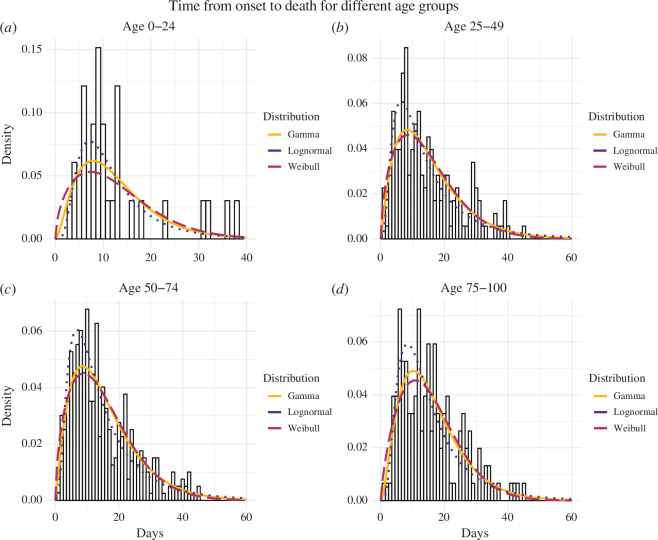
The probability density of the observed time between symptom onset and death is portrayed for four age groups: (*a*) 0–24 years, (*b*) 25–49 years, (*c*) 50–74 years and (*d*) 75–100 years, encompassing 36, 190, 426 and 164, respectively. These figures represent the proportion of the population captured in the survey. A comparative analysis of the delay distribution is then conducted across these distinct age groups. The probability density distributions of the gamma, Weibull and lognormal distributions illustrate the distribution of times from symptom onset to confirmation within each specific age group.

We conducted a comprehensive analysis of the duration between symptom onset and death, categorizing our findings by age groups, as presented in table 4. Remarkably, significant variations were observed in the meantime from symptom onset to death across these groups. For instance, the mean delay ranged from approximately 12.3 (95% CI: 9.35–16.8) days for the 0–24 years age group to 16.1 (95% CI: 15.5–16.0) days for the 75–100 years age group. The gamma distribution emerged as the most accurate representation for this duration across various age groups (25–49 years, 50–74 years and 75–100 years). Additionally, for the 0–24 years age group, the lognormal distribution demonstrated the best fit. The selection of both the lognormal and gamma distributions was validated by their lower AIC values compared with the gamma and Weibull distributions. For a detailed summary of our findings, please refer to table 4 and figure 6, which provide visual representations. Furthermore, we varied the date of symptom onset to compare our results with the interval censoring method, revealing slight differences as outlined in the electronic supplementary material, S2.4.

**Table 4.** Estimated distributions characterizing the time from symptom onset to death, for individuals of different ages. (The age groups are 0–24 years, 25–49 years, 50–74 years and 75–100 years, encompassing 36, 190, 426 and 164 cases, respectively; 95% credible intervals are shown in parentheses.)

gamma/age group	mean	shape	rate	AIC
0–24	12.59 (12.0–13.9)	2.77 (1.81–4.68)	0.22 (0.13–0.39)	223.6931
25–49	15.5 (14.8–16.3)	2.17 (1.79–2.68)	0.14 (0.11–0.18)	1261.345
50–74	15.7 (14.8–16.2)	2.21 (1.95–2.52)	0.14 (0.12–0.17)	2841.778
75–100	16.1 (15.5–16.9)	2.75 (2.24–3.42)	0.17 (0.14–0.22)	1076.022
Weibull/age group	mean	shape	scale	AIC
0–24	12.7 (10.0–15.6)	1.61 (1.23–2.15)	14.2 (10.8–17.7)	227.5161
25–49	14.7 (13.5–16.1)	1.58 (1.41–1.77)	16.4 (14.9–18.1)	1262.351
50–74	15.0 (14.1–15.8)	1.57 (1.46–1.70)	16.7 (15.6–17.8)	2847.374
75–100	15.5 (14.3–16.9)	1.87 (1.58–2.02)	17.5 (16.0–19.1)	1078.929
lognormal/age group	mean	mean-log	s.d.-log	AIC
0–24	12.3 (9.35–16.8)	2.33 (2.13–2.55)	0.60 (0.46–0.74)	219.1320
25–49	15.3 (12.8–18.2)	2.44 (2.32–2.55)	0.76 (0.68–0.84)	1276.453
50–74	15.3 (13.7–17.3)	2.46 (2.38–2.54)	0.74 (0.69–0.79)	2865.034
75–100	15.6 (13.5–18.3)	2.54 (2.44–2.65)	0.65 (0.58–0.72)	1084.599

### 3.5. Probability of death following hospitalization

**Figure 7.**
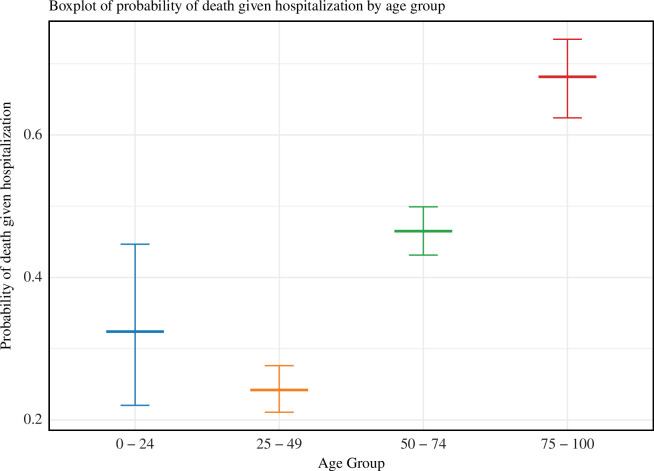
Probability of death given hospitalization in different age groups. For each age group, the central thick line depicts the median estimate, with the surrounding interval representing a 95% credible interval. The noticeable variation in these probabilities among different age groups underscores the importance of considering age-related differences in healthcare planning and interventions.

We estimated the probability that individuals who are hospitalized go on to die. For the 0–24 years and 25–49 years age groups, the probability of death given hospitalization is relatively low, with median estimates of 0.323 (95% CI: 0.220–0.446) and 0.241 (95% CI: 0.210–0.276), respectively. By contrast, in the 50–74 years age group, the probability of death given hospitalization is notably higher (0.465; 95% CI: 0.431–0.499). This signifies that individuals aged 50–74 years exhibit an increased vulnerability to adverse outcomes such as mortality when they require hospitalization. Finally, in the 75–100 years age group, the probability of death given hospitalization is at its highest at 0.681 (95% CI: 0.624–0.734). This finding underscores that individuals in this older demographic group face the most elevated risk of mortality following hospitalization. Boxplots representing the estimates of the probability of death following hospitalization in different age groups are shown in figure 7.

## 4. Discussion and conclusion

In this study, we used data from MERS-CoV cases in Saudi Arabia to estimate a range of epidemiological time intervals, including onset-to-admission, onset-to-confirmation, onset-to-reporting and onset-to-death. Specifically, we fitted different probability distributions to the data and selected the best-fitting distributions based on the AIC values. We found that epidemiological distributions can be sensitive to the age of the cases under consideration, highlighting the importance of considering age-related heterogeneities in epidemiological analyses. Furthermore, the precise parametric distribution that fitted the data best sometimes depended on the age group under consideration. A comprehensive understanding of the time intervals associated with MERS-CoV infections can contribute to informed policy decisions aimed at containment and the suppression of transmission.

The mean delay from symptom onset to hospitalization was shorter than the mean delay from symptom onset to reporting because only severe cases tend to be hospitalized. By contrast, all cases are reported, but those with mild symptoms may be reported with less urgency. After confirmation, information about each case is compiled and sent to the Saudi Arabia MoH, where it is processed and reported to the public within 1–2 days. This rapid reporting system ensures not only accuracy but also timeliness in disseminating vital information about an ongoing outbreak. Every confirmed case, whether the symptoms were severe or mild, was meticulously documented, confirmed and reported, enabling healthcare professionals and the public to stay updated on the disease’s progression and to take necessary precautions.

In addition to estimating epidemiological time intervals, we calculated the probability of death after hospitalization for different age groups. This provided insights into patients’ chances of recovery after being hospitalized based on their age. It is imperative to highlight the substantial variation observed between different age groups in the probability of death following hospitalization. This variation underscores the diverse risk profiles and outcomes experienced by individuals of varying ages when confronted with MERS-CoV. It is crucial that this is considered when planning targeted healthcare strategies, as it provides a nuanced understanding of the unique challenges and vulnerabilities specific to each age group. This comprehensive insight allows for the implementation of age-tailored interventions, ultimately enhancing the effectiveness of healthcare responses and ensuring a more efficient allocation of resources.

We further conducted a sensitivity analysis to compare our main results to analogous results in which interval-censored event times are accounted for. Specifically, interval censoring accounts for the range of different possible times at which events occur on the dates concerned. For example, if an individual develops symptoms on the day before they then report their infection, the true symptom onset to reporting delay could lie anywhere in the range 0–2 days (i.e. they could develop symptoms at the end of the first day and report at the beginning of the second day, or vice versa). We found our results to be very similar to whether or not interval censoring is accounted for.

As with any epidemiological analysis, our study does have some limitations. For example, recall bias may have affected our results. In particular, the recorded dates of symptom onset rely on patients’ recall after admission for MERS-CoV, which can introduce inaccuracies. In addition, in some analyses, we considered uncertainty in the dates of symptom onset. However, future analyses should also consider uncertainty in the dates of other events, such as hospitalization, confirmation, reporting and death dates. Finally, some patients were excluded from our analyses owing to incomplete clinical histories or missing characteristics, potentially leading to selection bias. Collection and analysis of additional data in the future would be useful to confirm the results of our study, as well as to explore heterogeneities that are not age-related.

In conclusion, we used a comprehensive and extensive nationwide database describing the characteristics of MERS-CoV patients to estimate a range of epidemiological periods. We fitted a range of parametric distributions to those data and demonstrated that there is substantial variation between individuals of different ages. We hope that the estimates obtained in our analysis will be useful for future modelling studies, as well as to inform effective public health policies to mitigate the negative impacts of MERS-CoV going forward.

## Data Availability

The MERS-CoV dataset is accessible upon direct request from the Ministry of HealthMoH of Saudi Arabia (https://od.data.gov.sa/en/request-dataset). For transparency and reproducibility, the code used in this analysis is available on GitHub at https://github.com/Yehyaalthobaity/MERS-COV_daley. Supplementary material is available online [19].
